# In Vitro Activities of Dithiocarbamate Derivatives against *Echinococcus multilocularis* Metacestode Vesicles

**DOI:** 10.3390/tropicalmed8120517

**Published:** 2023-12-12

**Authors:** Marc Kaethner, Georg Rennar, Tom Gallinger, Tobias Kämpfer, Andrew Hemphill, Patrick Mäder, Ana Luque-Gómez, Martin Schlitzer, Britta Lundström-Stadelmann

**Affiliations:** 1 Institute of Parasitology, Vetsuisse Faculty, University of Bern, 3012 Bern, Switzerland; marc.kaethner@unibe.ch (M.K.); tobias.kaempfer@unibe.ch (T.K.); andrew.hemphill@unibe.ch (A.H.); 2 Graduate School for Cellular and Biomedical Sciences, University of Bern, 3012 Bern, Switzerland; 3 Institute of Pharmaceutical Chemistry, Philipps-Universität Marburg, 35037 Marburg, Germanyschlitzer@staff.uni-marburg.de (M.S.); 4 Instituto de Síntesis Química y Catálisis Homogénea (ISQCH), Facultad de Ciencias, Universidad de Zaragoza-CSIC, 50009 Zaragoza, Spain; 5 Multidisciplinary Center for Infectious Diseases, University of Bern, 3012 Bern, Switzerland

**Keywords:** *Echinococcus*, platyhelminth, drug treatment, in vitro drug screening, disulfiram

## Abstract

The metacestode stage of the fox tapeworm *Echinococcus multilocularis* causes the severe zoonotic disease alveolar echinococcosis. New treatment options are urgently needed. Disulfiram and dithiocarbamates were previously shown to exhibit activity against the trematode *Schistosoma mansoni.* As both parasites belong to the platyhelminths, here we investigated whether these compounds were also active against *E. multilocularis* metacestode vesicles in vitro. We used an in vitro drug-screening cascade for the identification of novel compounds against *E. multilocularis* metacestode vesicles with disulfiram and 51 dithiocarbamates. Five compounds showed activity against *E. multilocularis* metacestode vesicles after five days of drug incubation in a damage marker release assay. Structure–activity relationship analyses revealed that a *S*-2-hydroxy-5-nitro benzyl moiety was necessary for anti-echinococcal activity, as derivatives without this group had no effect on *E. multilocularis* metacestode vesicles. The five active compounds were further tested for potential cytotoxicity in mammalian cells. For two compounds with low toxicity (Schl-32.315 and Schl-33.652), IC_50_ values in metacestode vesicles and IC_50_ values in germinal layer cells were calculated. The compounds were not highly active on isolated GL cells with IC_50_ values of 27.0 ± 4.2 µM for Schl-32.315 and 24.7 ± 11.5 µM for Schl-33.652, respectively. Against metacestode vesicles, Schl-32.315 was not very active either with an IC_50_ value of 41.6 ± 3.2 µM, while Schl-33.652 showed a low IC_50_ of 4.3 ± 1 µM and should be further investigated in the future for its activity against alveolar echinococcosis.

## 1. Introduction

Metacestodes of the fox tapeworm *Echinococcus multilocularis* are the causative agent of the severe zoonotic disease alveolar echinococcosis (AE). Humans and other accidental hosts acquire the infection via the oral uptake of eggs containing oncospheres. Upon the ingestion of *E. multilocularis* eggs, the parasite develops into the metacestode stage, which grows infiltratively into the liver of its host [1]. Metacestodes are fluid-filled cysts that are protected by an outer, acellular laminated layer rich in carbohydrates [2]. The interior side of this laminated layer is covered by a syncytial tegument and the germinal layer (GL) [3]. The GL contains undifferentiated stem cells, which give the parasite its high regenerative potential and have been suggested to be less affected by the current benzimidazole-based treatment against AE [4]. In addition, the GL is comprised of connective tissue, muscle cells, nerve cells, glycogen storage cells and subtegumentary cytons [5,6].

*E. multilocularis* is considered the first and third most important food-borne parasite in Europe and worldwide, respectively [7,8]. Human AE corresponds to a global burden of 688,000 disability-adjusted life years (DALYs) [9] and is an emerging disease [10,11]. Due to the cancer-like proliferative growth of metacestodes in the liver and the metastatic potential into other organs, AE is fatal if left untreated. Curative treatment options require surgical resection of all parasitic tissue, which is often not possible due to advanced disease progression [12]. Besides surgery, patients can be treated with the benzimidazoles (BMZs) albendazole or mebendazole [1]. While these BMZs have contributed to a drastic increase in the life expectancy of AE patients [13,14], they act only in a parasitostatic way and thus have to be taken lifelong. It is hypothesized that BMZs are not effective against the stem cells of *E. multilocularis* [15] and therefore treatment discontinuation leads to disease recurrence [16,17]. New treatment options against AE are urgently needed and should show efficacy against both the disease-causing metacestode stage as well as the stem cells.

Whole-organism-based screening plays an important role in the search for new antiparasitic drugs and is used to screen for active compounds against various helminths [18]. The repurposing of drugs that are licensed for the treatment of other diseases can reduce the time and costs of treatments to reach patients of neglected diseases including AE [19,20]. The drug disulfiram has been used in human patients to treat chronic alcoholism [21,22]. It inhibits human aldehyde dehydrogenases (ALDHs) [23] and thus leads to an accumulation of acetaldehyde and concomitant unpleasant symptoms like the flushing of skin, sweating, headache and nausea upon the ingestion of even small amounts of alcohol [24,25]. Disulfiram has been shown to be active in laboratory models against various parasites, such as the protozoans *Giardia lamblia* and *Leishmania major* [26,27], the nematode *Trichuris muris* [28], or the trematode *Schistosoma mansoni* [29,30,31]. However, disulfiram did not reach clinical treatment against any parasitosis. Interestingly, disulfiram has not been tested on *E. multilocularis*, nor any other cestodes.

Previously, disulfiram metabolites were used as a basis for the development of dithiocarbamate derivatives showing promising effects with increased activity against *S. mansoni* and reduced mammalian cell toxicity [30]. In the present study, we used an established in vitro drug-screening cascade [32] in order to test disulfiram and dithiocarbamate derivatives on metacestode vesicles, isolated GL cells of *E. multilocularis*, and performed a limited structure–activity relationship (SAR) analysis of these dithiocarbamates.

## 2. Materials and Methods

### 2.1. Chemicals and Reagents

All chemicals were purchased from Sigma-Aldrich (Buchs, Switzerland) and plastic ware was purchased from Sarstedt (Sevelen, Switzerland), unless stated otherwise. Dulbeccos’s modified Eagle medium (DMEM) and Penicillin and Streptomycin (10,000 Units/mL Penicillin, 10,000 μg/mL Streptomycin) were purchased from Gibco (Fisher Scientific AG, Reinach, Switzerland). Fetal bovine serum (FBS) and Trypsin/EDTA (0.05% Trypsin/0.02% EDTA) were purchased from Bioswisstec (Schaffhausen, Switzerland). Rat hepatoma (RH) cells (H-4-II-E) and human foreskin fibroblasts (HFFs) were from ATCC (Molsheim Cedex, France).

### 2.2. Synthesis of Dithiocarbamates

The synthesis of compounds was performed at the University of Marburg and by following the general routes published previously [30,33]. Structures, molecular weights, and activities against *E. multilocularis* metacestode vesicles are given in Table S1.

### 2.3. Mice and Ethics Statement

Strain maintenance of *E. multilocularis* H95 was performed in female BALB/c mice (Charles River Laboratories, Sulzheim, Germany) at the University of Bern. Mice were maintained in ventilated cages in a temperature-controlled room of 21 to 23 °C, a 12 h light/dark cycle and a relative humidity of 45 to 55%. Food and water were provided ad libitum and the cage was further enriched with a house (Tecniplast, Gams, Switzerland), nestlets (Plexx, Elst, The Netherlands) and a tunnel (Zoonlab, Castrop-Rauxel, Germany). Mice were intraperitoneally infected with metacestode material and euthanized two to four months post-infection as previously described [34]. All animals were treated in compliance with the Swiss Federal Protection of Animals Act (TSchV, SR455), and strain maintenance was approved by the Animal Welfare Committee of the canton of Bern under the license numbers BE30/19 and BE2/2022.

### 2.4. Overview of the General Screening Approach

**Figure 1:**
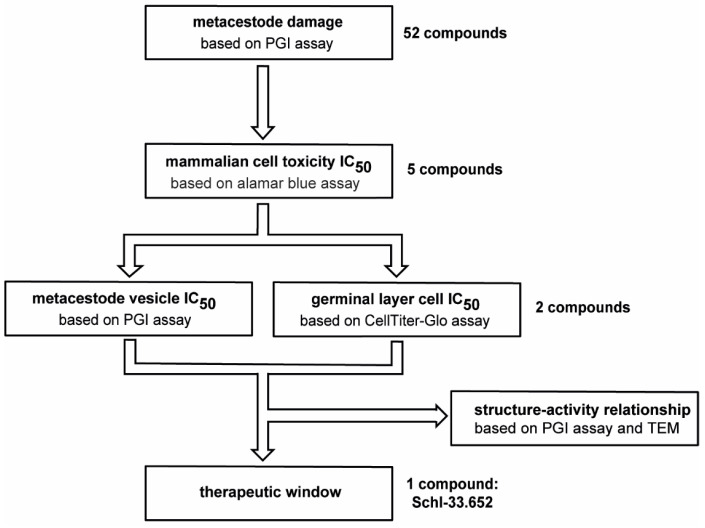
In total, 52 compounds were screened on *E. multilocularis* metacestode vesicles by PGI assay in an overview screen at 10 µM. Five active compounds were tested by alamar blue assay on mammalian cells to calculate IC_50_ values. Two compounds with low mammalian cell toxicity were tested on metacestode vesicles and GL cell cultures to determine IC_50_ values. The comparison of IC_50_ values on mammalian cells and on metacestode vesicles and GL cell cultures allowed us to define a potential therapeutic window for one compound (Schl-33.652) in vitro. Furthermore, structure–activity relationship for this and other active compounds was performed based on PGI assay and transmission electron microscopy (TEM).

In order to screen disulfiram and dithiocarbamate derivatives as potential new drug candidates, we used an in vitro screening cascade, in which the compounds were first screened against *E. multilocularis* metacestode vesicles via the phosphoglucose isomerase (PGI) assay, in which metacestode vesicle damage is assessed by the measurement of damage marker (PGI) release (Figure 1, see Section 2.6). Compounds that showed activity were then further tested in concentration series on pre-confluent and confluent RH cells and HFFs to evaluate toxicity on mammalian cells (see Section 2.7). Subsequently, compounds were assessed in concentration series on *E. multilocularis* metacestode vesicles via the PGI assay and on GL cell cultures via the CellTiter-Glo assay (see Section 2.9). The testing on mammalian cells as well as metacestode vesicles and GL cell cultures allowed the identification of a potential therapeutic window in vitro.

### 2.5. Cultivation of E. multilocularis Metacestode Vesicles

Metacestode vesicles were cultured at the University of Bern as described previously [34]. In short, metacestode vesicle material was resected from experimentally infected mice and pressed through a tea strainer (Migros, Berne, Switzerland). The material was incubated in PBS containing penicillin (100 U/mL), streptomycin (100 µg/mL), tetracycline (10 µg/mL) and levofloxacin (20 µg/mL) at 4 °C overnight. The next day, the parasite material was washed in PBS and co-cultured with semi-confluent RH cells in DMEM containing 10% FBS, penicillin (100 U/mL), streptomycin (100 µg/mL) and tetracycline (5 µg/mL). *E. multilocularis* metacestode vesicle material was then cultured at 37 °C in a humid, 5% CO_2_ atmosphere. Medium changes were performed once a week including the weekly addition of freshly trypsinized RH cells.

### 2.6. PGI Assay with E. multilocularis Metacestode Vesicles

PGI assays were conducted at the University of Bern as described previously [35,36]. In short, two-to-three-month-old metacestode vesicles of two to four mm in diameter were purified using 2% sucrose followed by several washing steps in PBS. Purified metacestode vesicles were mixed with two volumes of DMEM without phenol red containing penicillin (100 U/mL) and streptomycin (100 µg/mL). One mL of this mix of metacestode vesicles in medium was distributed to each well of a 48-well plate (Huberlab, Aesch, Switzerland). Compounds were first tested at 10 µM in triplicate using 0.1% of DMSO as a negative control and 0.1% Triton X-100 as a positive control. The metacestode vesicles were incubated under a humid, microaerobic atmosphere (85% N_2_, 10% CO_2_, 5% O_2_) [35] and supernatant samples were taken after five days. Samples were measured on an EnSpire multilabel reader (Perkin Elmer, Waltham, MA, USA). Shown are mean values and standard deviations (SDs). Compounds were considered active when they reached 20% of PGI release relative to Triton X-100. Active compounds were tested at concentrations of 90 to 0.4 µM in triplicate. Samples were measured on a HIDEX Sense microplate reader (Hidex, Turku, Finland). R studio version 4.3.0 was used to calculate IC_50_ values [37] and mean values and SDs are given for two independent experiments for the IC_50_ assessment. 

### 2.7. Cultivation and Cytotoxicity Assays with RH Cells and HFFs

The cultivation of RH cells and HFFs and the cytotoxicity assays were performed at the University of Bern as previously described [38], with a few modifications. RH cells and HFFs were cultivated in T175 flasks in DMEM containing 10% FBS, penicillin (100 U/mL), streptomycin (100 µg/mL) and tetracycline (5 µg/mL) in a humid, 5% CO_2_ incubator as described before [38]. For the cytotoxicity assay, compounds were tested on RH cells and HFFs grown as a confluent monolayer or pre-confluent cells. For the confluent setups, 50,000 RH cells or 10,000 HFFs were seeded per well in DMEM supplemented with 10% FBS, 100 U/mL penicillin, 100 μg/mL streptomycin and 5 μg/mL tetracycline, while for the pre-confluent setups, 5000 RH cells or 1000 HFFs were seeded. The drugs were added in 1:3 serial dilutions with final concentrations from 90 µM to 0.1 µM in triplicate or the respective amount of DMSO as negative control. The cells were incubated for five days at 37 °C and 5% CO_2_ in a humid atmosphere. Resazurin was added to a final concentration of 10 mg/L in order to measure cell viability as described before [39]. The plates were measured on a HIDEX Sense microplate reader directly after the addition of the resazurin solution and after 50 min of incubation at RT in the dark. Cell viability was calculated using the difference from both time points and set relative to the respective DMSO controls (0.3% or 0.1%). IC_50_ values were calculated in R studio version 4.3.0 [37] and mean values and SDs of three independent experiments are given. Datapoints indicating an IC_50_ higher than the highest concentration tested (>90 µM) were excluded.

### 2.8. Isolation of GL Cells of E. multilocularis 

The isolation of GL cells was performed at the University of Bern according to the updated protocol recently published [35]. In short, DMEM containing 10% FBS, penicillin (100 U/mL), streptomycin (100 µg/mL) and tetracycline (5 µg/mL) was incubated with RH cells in 50 mL medium. Medium in which 10^6^ cells were incubated for six days and 10^7^ cells we incubated for four days was mixed 1:1. It was sterile-filtered and used as conditioned medium (cDMEM). In vitro grown metacestode vesicles of at least six months were cleaned of RH cells by an incubation step in distilled water. Then, mechanically broken metacestode vesicle tissue was incubated in eight volumes of Trypsin/EDTA solution at 37 °C for 30 min. GL cells were collected by filtering the supernatant through a 30 µM mesh (Sefar AG, Heiden, Switzerland), separated from calcareous corpuscles via a short centrifugation step (50× *g*, 30 s, RT) and pelleted at 600× *g* for 10 min. The cells were resuspended in cDMEM and quantified as arbitrary units (AUs) via measuring the OD_600_ value of a 1:100 diluted cell solution. An OD_600_ value of 0.1 of a 1:100 diluted cell solution corresponded to one AU of the undiluted cell solution. 

### 2.9. GL Cell Viability Assay

GL cell viability upon treatment with Schl-32.315 and Schl-33.652 was assessed at the University of Bern as described previously [35]. In short, 15 AU GL cells were distributed in 12.5 µL cDMEM in wells of a black 384-well plate and compounds were added in 12.5 µL cDMEM resulting in final concentrations from 90 to 0.1 µM and respective DMSO concentrations of 0.1% and 0.3% as a control. Each concentration was tested in quadruplicate and the cells were incubated under microaerobic conditions for five days. Cell viability was measured upon the addition of 25 µL of CellTiter-Glo (Promega, Dübendorf, Switzerland) containing 1% Triton X-100 on a HIDEX Sense microplate reader. IC_50_ values were calculated for each of three independent experiments in R studio version 4.3.0 [37] and mean and SD values are given. 

### 2.10. Transmission Electron Microscopy

Transmission electron microscopy (TEM) was carried out with *E. multilocularis* metacestode vesicles treated with the most active compound Schl-33.652, the inactive derivative Schl-33.290 without a *S*-2-hydroxy-5-nitro benzyl residue, and the respective concentration of DMSO. Compounds were tested at 10 µM concentration and samples were taken after five days of incubation under microaerobic conditions as described above (Section 2.6). Processing of the samples was carried out according to the adapted protocol described previously [35]. In short, metacestode vesicles were fixed in 100 mM sodium cacodylate at pH 7.3 containing 2% glutaraldehyde at 4 °C overnight, subsequently washed three times in 100 mM sodium cacodylate, and post-fixed in 100 mM sodium cacodylate containing 2% osmium tetroxide at RT for 90 min. The samples were washed three times in water and then dehydrated by washing steps in increasing concentrations of ethanol (30%, 50%, 70%, 90% and three times with 100%). The dehydrated samples were embedded in Epon 812 resin and incubated at 37 °C. Over 2.5 h, the resin was changed two times. After the last resin change, the samples were incubated for 24 h at RT and subsequently polymerized by an incubation step at 65 °C overnight. An ultramicrotome (Reichert and Jung, Vienna, Austria) was used to cut 80 nm sections of the samples, and then these sections were loaded onto formvar-carbon-coated nickel grids (Plano GmbH, Marburg, Germany). Finally, the specimens were stained with Uranyless™ and lead citrate (Electron Microscopy Sciences, Hatfield, PA, USA) and then photographed on an FEI Morgagni transmission electron microscope (Field Electron and Ion Company, Hillsboro, OR, USA) operated at 80 kV.

## 3. Results

**Figure 2:**
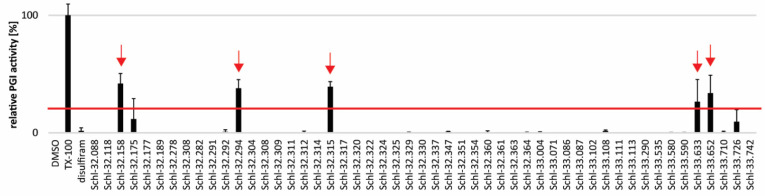
Disulfiram and 51 dithiocarbamates were tested on *E. multilocularis* metacestode vesicles at 10 µM and samples were taken after five days of drug incubation. PGI activity was calculated relative to the positive control 0.1% Triton X-100. The experiment was performed in triplicate and shown are mean values and SDs. The red line indicates the 20% cut-off.

In order to test for potential anti-echinococcal activity of disulfiram and dithiocarbamate derivatives, we assessed these 52 compounds at 10 µM on *E. multilocularis* metacestode vesicles in vitro (Figure 2, Table S1). The five compounds Schl-32.158, Schl-32.294, Schl-32.315, Schl-33.633 and Schl-33.652 were active after five days with relative PGI activities of 41.9 ± 8.5%, 37.9 ± 7.3%, 39.2 ± 4.3%, 26.4 ± 18.9% and 33.7 ± 15.3%, respectively. 

As a first step towards analyzing a potential therapeutic window of the five active compounds, we tested them for their cytotoxicity on HFF and RH cells in vitro (Table 1). 

Schl-32.158 and Schl-32.294 were toxic to pre-confluent RH cells with IC_50_ values < 15 µM. Schl-33.633 was slightly more toxic on pre-confluent and confluent RH cells, as well as confluent HFFs. The two least toxic compounds, Schl-32.315 and Schl-33.652, were further investigated and IC_50_ values were assessed on *E. multilocularis* metacestode vesicles (Table 2). The IC_50_ values were 41.6 ± 3.2 µM for Schl-32.315 and 4.3 ± 1 µM for Schl-33.652, respectively. Subsequently, both compounds were also tested on isolated GL cells of this parasite (Table 2). Here, the calculated IC_50_ values of Schl-32.315 and Schl-33.652 were 27 ± 4.2 µM and 24.7 ± 11.5 µM, respectively.

**Figure 3:**
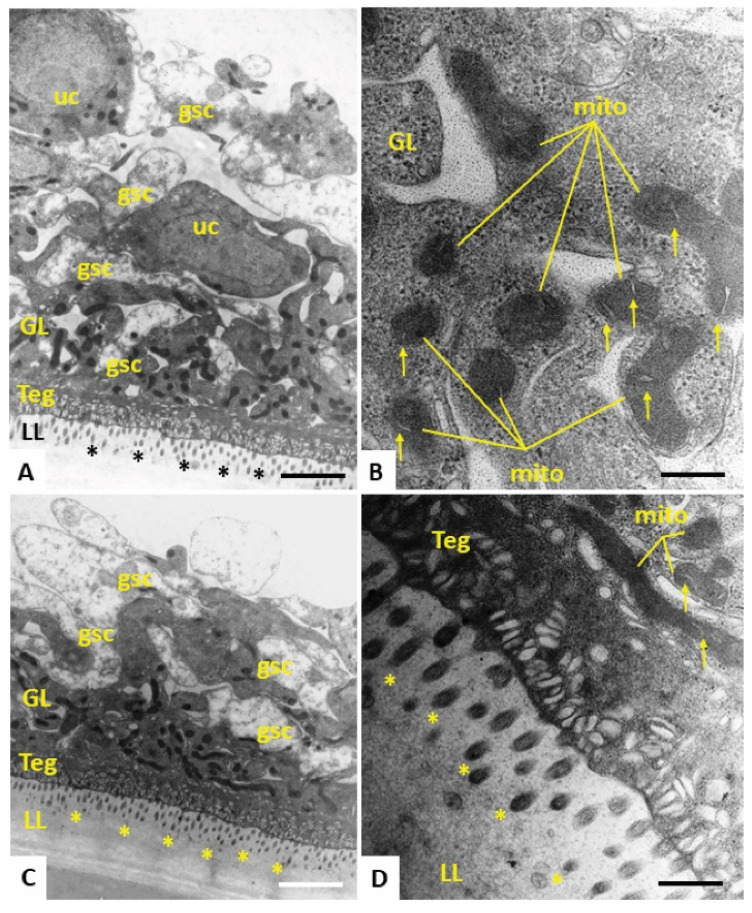
(**A**,**C**) Lower magnification views of the metacestode vesicle wall. The outer metacestode vesicle surface is represented by the carbohydrate-rich laminated layer (LL), followed by the syncytial tegument (Teg) and the germinal layer (GL), the latter of which contains numerous irregularly shaped and electron-dense mitochondria. Undifferentiated cells (uc) and glycogen storage cells are also discernible. (**B**) Higher-magnification view of the GL with electron-dense mitochondria (mito), with cristae present in the mitochondrial matrix (indicated by arrows). (**D**) Teg–LL interface with microtriches (marked in A, C and D with *) protruding well into the LL. Bars in **A** = 2.34 µm, **B** = 0.3 µm, **C** = 2.34 µm, **D** = 0.47 µm.

**Figure 4:**
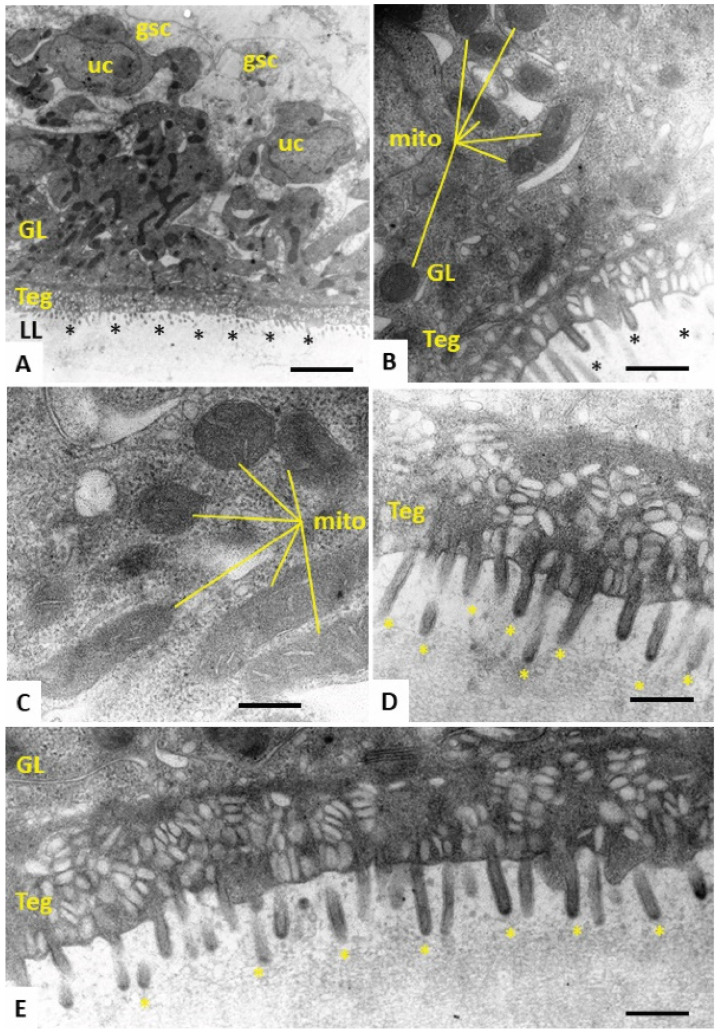
(**A**) Section through the metacestode vesicle wall, with the outer laminated layer (LL), tegument (Teg) and the germinal layer (GL). Undifferentiated cells (uc), glycogen storage cells (gsc) and microtriches marked with * are also shown. A high number of irregularly shaped and electron-dense mitochondria are seen in the GL. Mitochondria (mito) are shown at higher magnification in (**B**,**C**), with cristae embedded in the electron-dense matrix. (**D**,**E**) Higher-magnification views of the tegument–LL interface with a high number of microtriches (*) protruding into the LL, which is largely devoid of vesicular inclusions. Bars in **A** = 2.8 µm, **B** = 0.45 µm, **C** = 0.35 µm, **D** = 0.3 µm and **E** = 0.35 µm.

**Figure 5:**
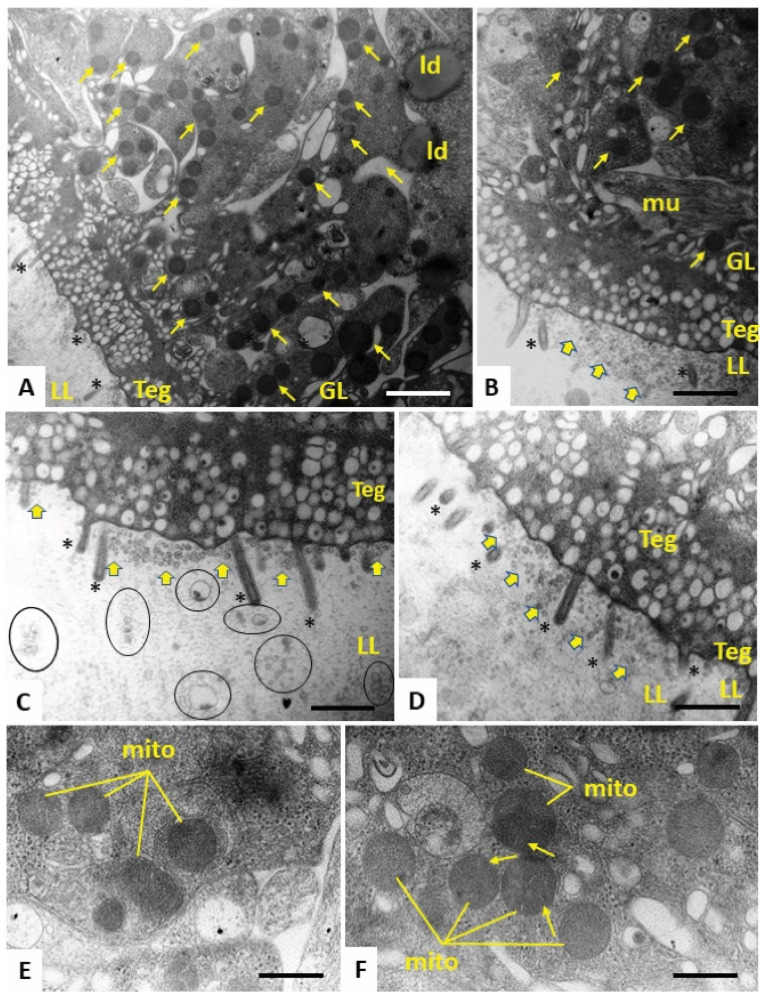
(**A**) Low-magnification view through the metacestode vesicle wall, with laminated layer (LL), tegument (Teg) and germinal layer (GL). Note the drastically reduced number of microtriches (*), as well as the appearance of rounded electron-dense mitochondria (small arrows) and lipid droplets (ld) in the GL. Higher magnification (**B**–**D**) shows that the LL–Teg interface is filled with small vesicles that are seemingly released into the LL (yellow arrows). In (**B**), a normally shaped muscle cell (mu) is shown. In (**C**), larger vesiculated structures (marked with circles) can be seen to be embedded in the more distal portions of the LL. (**E**,**F**) Higher-magnification views of rounded mitochondria present in the LL, with cristae only occasionally clearly displayed (arrows). Bars in **A** = 1.4 µm, **B** = 0.8 µm, **C** = 0.7 µm, **D** = 0.7 µm and **E** and **F** = 0.3 µm.

In order to study the morphological alterations of *E. multilocularis* metacestode vesicles caused upon treatment with the most active compound Schl-33.652 in comparison to the inactive derivative Schl-33.290 (without a *S*-2-hydroxy-5-nitro benzyl residue), or the respective amount of DMSO, we performed TEM after five days of drug incubation. In control samples treated with DMSO only, the metacestode vesicle tissue exhibited mitochondria of various shapes and sizes, with few cristae detectable in some instances. At the interface of the tegument and laminated layer, a high number of microtriches were seen protruding into the matrix of the laminated layer (Figure 3). Identical features were detected in metacestode vesicles upon treatment with the inactive compound Schl-33-290 (Figure 4). However, upon treatment with the active compound Schl-33.652 (Figure 5), alterations could be seen with respect to the overall shape of the mitochondria, as they displayed a rather uniform size and round shape. In addition, the number of microtriches protruding from the tegument into the laminated layer was markedly reduced, and a high density of extracellular vesicles could be observed within the laminated layer in the close vicinity of the tegument and microtriches. Furthermore, larger vesiculated structures were frequently seen to be embedded in the more distal portions of the LL as well. 

## 4. Discussion

Whole-organism-based screening has been frequently used in anthelmintic drug discovery [18]. For platyhelminths, various studies have been reported on whole-organism-based in vitro screenings of compound series, and structure–activity relationship studies in order to understand compound activity and further improve selected molecules. Examples are derivatives of quinoxaline and tetraazamacrocyclic compounds, disulfiram and dithiocarbamates that were tested on the trematode *S. mansoni* [30,40,41], or quinoxaline 1,4-di-N-oxide derivatives on the trematode *Fasciola hepatica* [42]. Also on cestodes, similar studies have been conducted, for example on derivatives of mefloquine, nitazoxanide and di-N-aryl-diguanidino compounds on *E. multilocularis* [34,36,43]. In the present study, we tested disulfiram and 51 dithiocarbamate derivatives (previously studied on *S. mansoni* [30]) on *E. multilocularis* metacestode vesicles. Five dithiocarbamate derivatives were active, but interestingly, disulfiram showed no effect in the metacestode damage assay based on PGI detection. This was unexpected, as disulfiram was active against a variety of protozoan parasites, nematodes and trematodes [28,31,44]. 

Of the 51 dithiocarbamate derivatives tested on *E. multilocularis* metacestode vesicles, five compounds (Schl-32.158, Schl-32.294, Schl-32.315, Schl-33.633 and Schl-33.652) displayed activity, and all contain a *S*-2-hydroxy-5-nitro benzyl residue. This moiety seems to be essential for activity as compounds with the same core structure without this specific residue (Schl-32.088, Schl-32.291, Schl-32.314, Schl-33.071 and Schl-33.290) were not active in the PGI assay. This was further confirmed by a comparison of Schl-33.652 and Schl-33.290 via TEM. However, the *S*-2-hydroxy-5-nitro benzyl moiety was not the only requirement for activity, as several compounds (Schl-32.280, Schl-32.308, Schl-32.322, Schl-33.004, Schl-33.086, Schl-33.087, Schl-33.102, Schl-33.108, Schl-33.111, Schl-33.113, Schl-33.535, Schl-33.580, Schl-33.726 and Schl-33.742) that also contain this substructure were not active. Further SAR analyses revealed that in the active compounds Schl-33.633 and Schl-33.652, an *N*-sulfonyl piperazine and an amidosulfonyl piperazine, respectively, are present. However, the presence of these moieties is neither necessary (Schl-32.158, Schl-32.294 and Schl-32.315) nor sufficient (Schl-33.004, Schl-33.102, Schl-33.108, Schl-33.111, Schl-33.535 and Schl-33.726) to yield active compounds in conjunction with the *S*-hydroxy-nitro-benzyl moiety. Small deviations from the active structure were not tolerated as the replacement of the cyclohexyl residue by methyl (Schl-33.004), cyclopropyl (Schl-33.102), or phenyl (Schl-33.108) abolished activity. However, when the cyclohexyl residue was replaced with thiomorpholino, the resulting sulfuric acid diamide Schl-33.652 was also active. While the cationic piperazinyl derivative Schl-32.322 was not active, its *tert*-butyloxycarbonyl (Boc) derivative Schl-32.315 showed activity. Most probably, the neutral Boc derivative, in contrast to its charged counterpart, is able to penetrate membranes, leading to enhanced activity.

Therefore, at this point, it can only be concluded that the presence of a 2-hydroxy-5-nitrobenzyl substituent at the dithiocarbamate sulfur is a necessary, but not sufficient, structural prerequisite for dithiocarbamate compounds active against *E. multilocularis*.

The two dithiocarbamates Schl-32.315 and Schl-33.652 that showed the lowest toxicity in mammalian RH cells and HFFs were further tested on *E. multilocularis* metacestode vesicles and GL cells using concentration series to calculate IC_50_ values. Both compounds showed IC_50_ values in GL cells that were in the same range as in pre-confluent RH cells, and IC_50_ values were two- to three-fold lower than in confluent RH and HFF cells. Interestingly, Schl-33.652 showed a low IC_50_ value against *E. multilocularis* metacestode vesicles at concentrations that were 6- to 17-fold lower compared to RH cells or 16- to 19-fold lower compared to HFFs, depending on the confluency of the cells. Thus, based on in vitro data, the compound Schl-33.652 shows a putative therapeutic window, albeit not exhibiting strong parasiticidal activity against GL cells, but nevertheless should be further evaluated against *E. multilocularis* in mouse models of AE [45,46]. 

We performed TEM with *E. multilocularis* metacestode vesicles in order to study which ultrastructural alterations were caused by Schl-33.652 to obtain an initial understanding of the mode of action of this compound. Interestingly, Schl-33.290, which was inactive in the PGI assay, did not cause structural changes, and parasites exhibited structural features that were identical to the DMSO control. Treatments of metacestode vesicles with Schl-33.652 resulted in distinct changes in mitochondrial shapes and sizes, treatment was associated with a profoundly reduced number of microtriches embedded in the laminated layer, and these microtriches were closely associated with a high density of extracellular vesicles that accumulated at the laminated layer–tegumental border. No other ultrastructural changes were noted. Thus, it is not clear to what extent Schl-33.652 affects the viability of these metacestode vesicles. In this respect, it would be very interesting to further investigate whether this compound impairs the functional activity of mitochondria by employing assays to monitor mitochondrial respiration of *E. multilocularis* GL cells [38]. The formation of extracellular vesicles, putatively caused by increased secretory activity, has also been reported with nitazoxanide treatment of *E. multilocularis* metacestode vesicles [47]. However, the potential mechanism for this is not known. The results we obtained from TEM are in accordance with the results from the PGI assay, in which Schl-33.652, but not Schl-33.290, showed activity against *E. multilocularis* metacestode vesicles. However, besides the reduced number of microtriches, no loss of structural integrity of the tegument could be seen by TEM. Thus, it is possible that the increased PGI activity could have been caused by increased secretory activity rather than a direct physical impact on the tegumental integrity.

Further experiments analyzing the SAR of Schl-33.652 and the other active compounds would be very interesting in order to generate a new series of compounds with potentially increased anti-echinococcal activity and decreased mammalian cell toxicity. Also, of the 51 tested dithiocarbamate derivatives, Schl-32.158 was the only compound with a 2-hydroxy-5-nitrobenzyl group that was not a piperazine derivative. Further exploration of the SAR of this new class of compounds may lead to improved potential drugs. 

The present study analyzed the effects of dithiocarbamate structures on *E. multilocularis* metacestode vesicles and GL cells and suggests that this structural family has the potential to provide new drug candidates against AE.

## Figures and Tables

**Table 1:** Cytotoxicity of five dithiocarbamates on HFF and RH cells. Three independent experiments were performed and shown are mean IC_50_ values and SDs.

Compound	HFF Pre-Confluent IC_50_	HFF Confluent IC_50_	RH Pre-Confluent IC_50_	RH Confluent IC_50_
Schl-32.158	30.4 ± 5.7 µM	44.8 ± 8.8 µM	11 ± 0.7 µM	59.7 ± 9.4 µM
Schl-32.294	38.2 ± 5.9 µM	55 ± 16.2 µM	14.6 ± 1.9 µM	55.4 ± 19.6 µM
Schl-32.315	42.9 ± 3.3 µM	78.9 ± 5.5 µM	29.8 ± 4.3 µM	58.6 ± 19.2 µM
Schl-33.633	53.5 ± 16.5 µM	72.4 ± 11.6 µM	24.3 ± 6 µM	50 ± 10.9 µM
Schl-33.652	69.8 ± 27.4 µM	83.2 ± 0 µM	26.6 ± 11 µM	74.9 ± 13.6 µM

**Table 2:** Shown are mean IC_50_ values and SD of two independent experiments with metacestode vesicles and three independent experiments with GL cells.

Compound	Metacestode Vesicle IC_50_	GL Cell IC_50_
Schl-32.315	41.6 ± 3.2 µM	27.0 ± 4.2 µM
Schl-33.652	4.3 ± 1 µM	24.7 ± 11.5 µM

## Data Availability

All data represented in this study is available within the manuscript.

## Supplementary Materials

The following supporting information can be downloaded at: https://www.mdpi.com/article/10.3390/tropicalmed8120517/s1, Table S1: Structures and activity of disulfiram and dithiocarbamate derivatives.
