# Supplementary material for: In Vitro Activities of Dithiocarbamate Derivatives against Echinococcus multilocularis Metacestode Vesicles
Source: Trop Med Infect Dis. 2023 Dec 12;8(12):517. doi: 10.3390/tropicalmed8120517 (PMC10747736; doi:10.3390/tropicalmed8120517)
Supplement: Supplementary file 1 [file tropicalmed-08-00517-s001.zip › tropicalmed-2752313-supplementary.pdf]

**Table S1. Structures and activity of disulfiram and dithiocarbamate derivatives.**

| Compound | Structure | Molecular weight [g/mol] | PGI assay |
|---|---|---|---|
| Disulfiram | 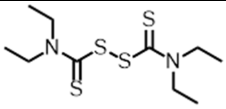 | 296.54 | Not active |
| Schl-32.088 | 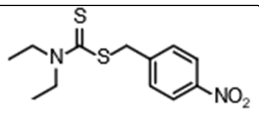 | 284.39 | Not active |
| Schl-32.118 | 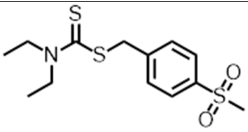 | 317.49 | Not active |
| Schl-32.158 | 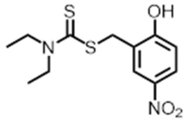 | 300.39 | **Active** |
| Schl-32.175 | 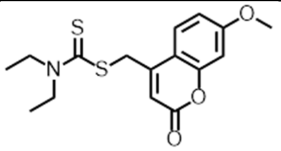 | 337.46 | Not active |
| Schl-32.177 | 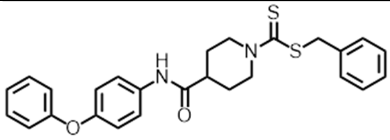 | 462.63 | Not active |
| Schl-32.189 | 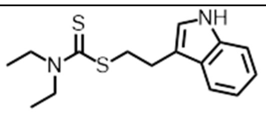 | 292.46 | Not active |
| Schl-32.278 | 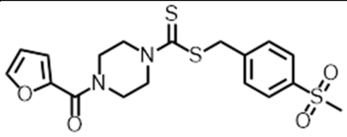 | 424.56 | Not active |
| Schl-32.280 | 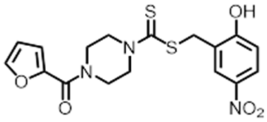 | 407.46 | Not active |
| Schl-32.282 | 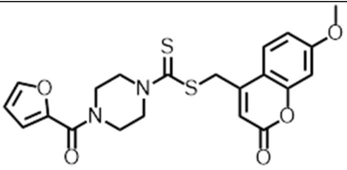 | 444.52 | Not active |
| Schl-32.291 | 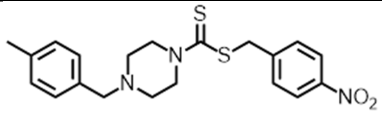 | 401.54 | Not active |
| Schl-32.292 | 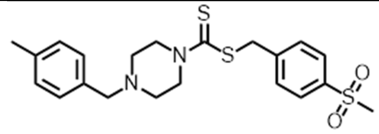 | 434.64 | Not active |

| | | | |
|---|---|---|---|
| Schl-32.294 | 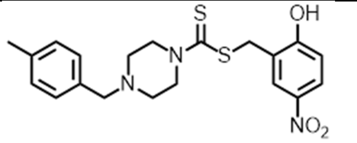 | 417.54 | **Active** |
| Schl-32.304 | 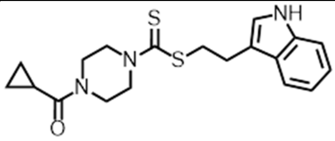 | 373.54 | Not active |
| Schl-32.308 | 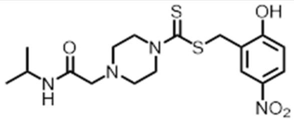 | 412.52 | Not active |
| Schl-32.309 | 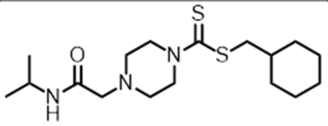 | 357.58 | Not active |
| Schl-32.311 | 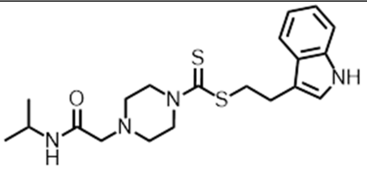 | 404.59 | Not active |
| Schl-32.312 | 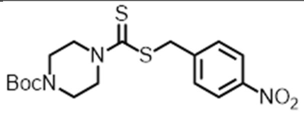 | 397.51 | Not active |
| Schl-32.314 | 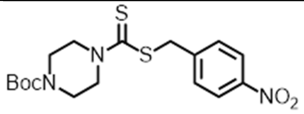 | 377.52 | Not active |
| Schl-32.315 | 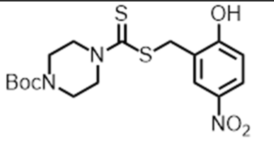 | 413.51 | **Active** |
| Schl-32.317 | 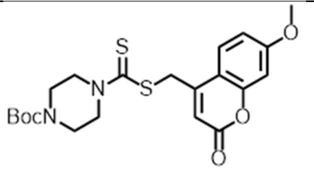 | 450.57 | Not active |
| Schl-32.320 | 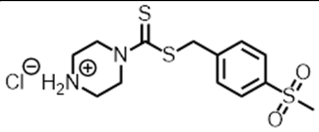 | 366.95 | Not active |
| Schl-32.322 | 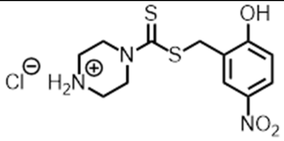 | 349.85 | Not active |
| Schl-32.324 | 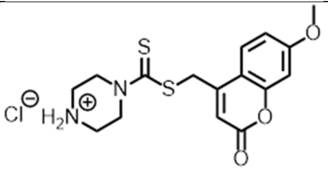 | 386.92 | Not active |
| Schl-32.325 | 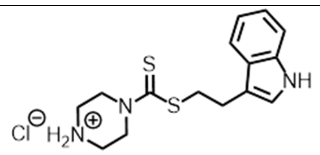 | 341.92 | Not active |

| | | | |
|---|---|---|---|
| Schl-32.329 | 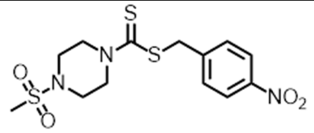 | 375.49 | Not active |
| Schl-32.330 | 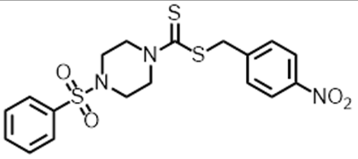 | 437.56 | Not active |
| Schl-32.337 | 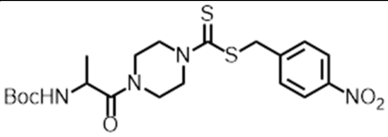 | 468.59 | Not active |
| Schl-32.347 | 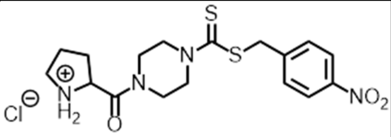 | 430.97 | Not active |
| Schl-32.351 | 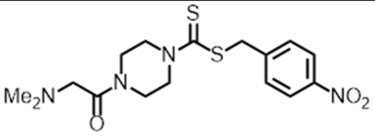 | 382.50 | Not active |
| Schl-32.354 | 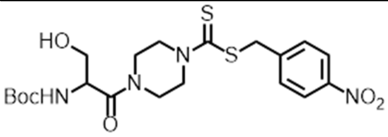 | 484.59 | Not active |
| Schl-32.360 | 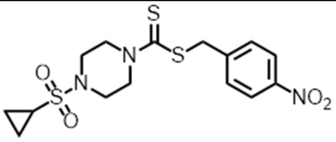 | 401.52 | Not active |
| Schl-32.361 | 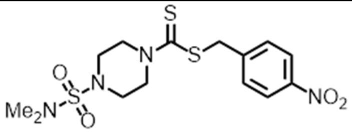 | 404.53 | Not active |
| Schl-32.363 | 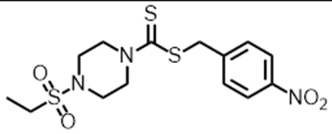 | 389.51 | Not active |
| Schl-32.364 | 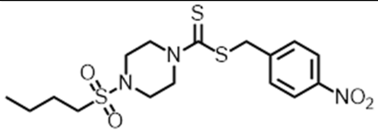 | 417.57 | Not active |
| Schl-33.004 | 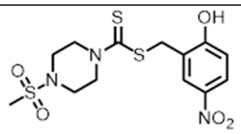 | 391.5 | Not active |
| Schl-33.071 | 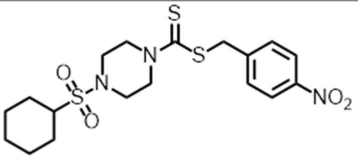 | 443.6 | Not active |
| Schl-33.086 | 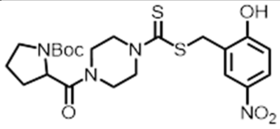 | 510.62 | Not active |

| | | | |
|---|---|---|---|
| Schl-33.087 | 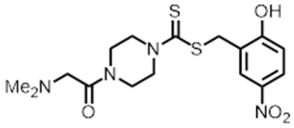 | 512.52 | Not active |
| Schl-33.102 | 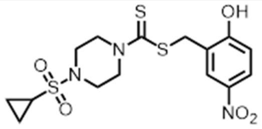 | 417.51 | Not active |
| Schl-33.108 | 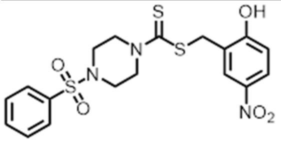 | 453.55 | Not active |
| Schl-33.111 | 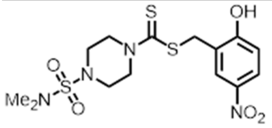 | 420.52 | Not active |
| Schl-33.113 | 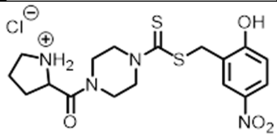 | 524.53 | Not active |
| Schl-33.290 | 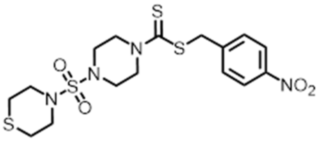 | 462.62 | Not active |
| Schl-33.535 | 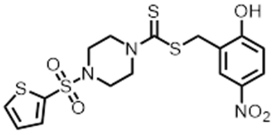 | 459.57 | Not active |
| Schl-33.580 | 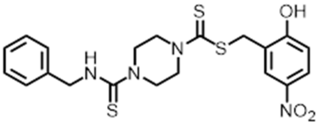 | 462.60 | Not active |
| Schl-33.590 | 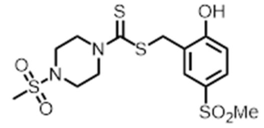 | 424.56 | Not active |
| Schl-33.633 | 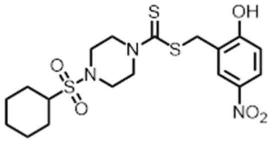 | 459.59 | **Active** |
| Schl-33.652 | 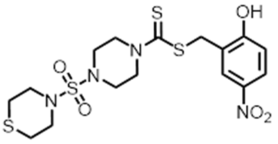 | 478.62 | **Active** |
| Schl-33.710 | 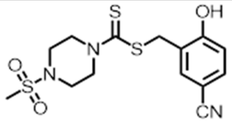 | 371.49 | Not active |
| Schl-33.726 | 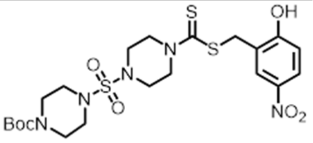 | 561.69 | Not active |

| | | | |
|---|---|---|---|
| Schl-33.742 | 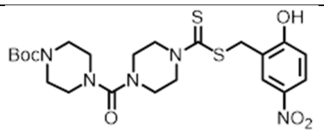 | 525.64 | Not active |
